# Supplementary material for: Structural insights into scaffold-guided assembly of the Pseudomonas phage D3 capsid
Source: Nat Commun. 2025 Nov 23;16:11586. doi: 10.1038/s41467-025-66648-1 (PMC12749128; doi:10.1038/s41467-025-66648-1)
Supplement: Supplementary file 1 — Supplementary information [file 41467_2025_66648_MOESM1_ESM.pdf]

Structural insights into scaffold-guided assembly of the Pseudomonas phage D3 capsid

Supplementary Information

Anna K. Belford, Joshua B. Maurer, Robert L. Duda, Alexis Huet* and James F. Conway*

Department of Structural Biology, University of Pittsburgh School of Medicine, Pittsburgh PA, USA.

*Co-Corresponding authors: James F. Conway, james.conway@pitt.edu
Alexis Huet, alh141@pitt.edu

This PDF file includes:

Supplementary Methods
Supplementary Tables S1 to S5
Supplementary Figures S1 to S12

Supplementary Methods

Short Descriptions (SMS#)

Supplementary Method 1.

Buffers Related to this Study

Lysis buffer:	50 mM Tris-HCl pH 8.0, 5 mM EDTA
D3 P1 Lysis buffer:	50 mM Tris-HCl pH 6.8, 100 mM K Glutamate, 10 mM EDTA
G buffer:	20 mM Tris-HCl pH 7.5, 100 mM NaCl
D3 P1 buffer:	50 mM Tris/50 mM Bis-Tris Propane-HCl pH 6.0, 100 mM potassium glutamate
Dialysis buffer:	5 mM Tris-HCl pH 7.5
D3 P1 Dialysis buffer:	2.5 mM Tris/2.5 mM Bis-Tris Propane pH 6.0
TAMg buffer:	40 mM Tris-HCl, 20 mM acetic acid pH 8.1, 1 mM $MgSO_4$

Supplementary Method 2.

Creation of expression plasmids and strains containing phage D3 capsid genes. Phage D3 was obtained from Andrew Kropinski. pT7-5[62] was used as the expression plasmid for D3 capsid genes. Plasmids used for expression contained the T7 promoter, D3 capsid genes 4, 5, and/or 6, and an ampicillin resistance gene. Plasmids were created by cut-and-paste digestion, purification, and ligation; site directed mutagenesis in which 2 of 4 primers are phosphorylated to allow for ligation and reamplification,[63] Klenow DNA polymerase I fill-in (Boehringer Mannheim, Ingelheim, Germany); and PCR amplification with pfu (and later phusion) PCR kits (New England Biolabs, Ipswich, MA) (ST2 & SMD1). Created plasmids were co-transformed with pLysS into BL21(DE3) by electroporation (SMD2).

Supplementary Method 3.

Production of Proheads from Plasmids.

Proheads were purified through PEG precipitation, differential sedimentation, velocity centrifugation with glycerol gradients, and anion exchange chromatography. BL21(DE3) pLysS strains containing expression plasmids with phage D3 capsid genes (ST3) were grown and expressed under autoinduction conditions.[60] Cells were harvested by centrifugation, resuspended in lysis buffer, and lysed by adding 0.25% (w/v) Triton X-100. Viscosity was reduced by treating with 25 μg/mL deoxyribonuclease I and 12.5 mM $MgSO_4$. The lysate was clarified by centrifugation and precipitated with 6% (w/v) PEG 8000/0.5 M Salt (potassium glutamate for P1; NaCl for P2). Precipitate was pelleted by centrifugation, resuspended in buffer (D3 P1 buffer for P1; G for P2), and repelleted by ultracentrifugation in Ti-45 (Beckman Coulter, Brea, CA) at 35000 rpm for 2 hours. Pellet was resuspended, loaded onto 10-30% glycerol gradients in same buffer, and sedimented in SW32Ti (Beckman) for 105 minutes at 29000 rpm. The prohead band was extracted, dialyzed, ran over an anion exchange column (Applied Biosystems, Waltham, MA), and pelleted again in Ti-45 for 2 hours at 35000 rpm. Pellet was resuspended in a small amount of buffer.

Supplementary Method 4.

Small Scale Expression of Capsid Genes from Plasmids

Expression of plasmids under autoinduction conditions [60] allows for rapid evaluation of plasmid expression phenotypes of capsid genes from (ST2) when performed small scale as compared to large-scale preps (1.5 mL vs. 300 mL).[64] After growth to high density, cells were lysed with Triton X-100, treated with DNase I, and separated by centrifugation into cell pellet and cell lysate. The cell lysate was separated

into two fractions, one of which was PEG precipitated, pelleted, and resuspended. The three fractions of cell pellet (PT), cell lysate (SUP), and PEG pellet (PEG) were analyzed by native agarose and SDS polyacrylamide gel electrophoresis.

Supplementary Method 5.

Gel Electrophoresis.

Native Agarose Gel Electrophoresis

Agarose gel electrophoresis was performed in submarine gel boxes (Hoeffer, Holliston, MA) using TAMg buffer. Samples were loaded with a dye/glycerol loading solution (50% (v/v) glycerol, 0.025% (w/v) bromophenol blue, 0.025% (w/v) xylene cyanol XFF) and run at ~120 V on agarose gels (usually 1.2% (w/v)). Gels were stained with ~0.002% (w/v) Coomassie Brilliant Blue R250 in ~4.5% (v/v) methanol, ~9.9% acetic acid, then destained with 10% acetic acid, and photographed wet.

Samples were TCA (trichloroacetic acid) precipitated prior to sodium dodecyl sulfate (SDS) polyacrylamide gel electrophoresis to prevent spontaneous crosslinking of D3 major capsid protein during sample preparation. Precipitates were washed with acetone, suspended in SDS sample buffer, heated in a boiling bath for 2.5 min, and run on 12% SDS acrylamide minigels. The original buffers described by Laemmli[65] were used. A low-crosslinker recipe (33.5% (w/v) acrylamide + 0.3% (w/v) methylene bisacrylamide)[66] was used. Gels were stained with 0.5% (w/v) Coomassie Brilliant Blue R250 in 50% (v/v) Methanol and 10% acetic acid, destained with 10% acetic acid, and photographed wet.

Supplementary Methods – Detailed Descriptions (SMD#)

Supplementary Method 1.

Inserting Phage D3 Capsid Proteins into pT7-5 Plasmid

Phage D3 was obtained from Andrew Kropinski. D3 capsid proteins were PCR amplified from phage genome in 50 μL volume reactions using pfu (and later phusion) protocol (New England Biolabs, Ipswich, MA) with the primers listed in (ST1) and methods in (ST2). Vectors and inserts were digested with restriction enzymes (New England Biolabs) and ligated with T4 ligase (New England Biolabs). Site directed mutagenesis was achieved by phosphorylating 2 of 4 primers to allow ligation and amplification.[63] Ligated plasmids were transformed into electrocompetent DH10B cells.

Supplementary Method 2.

Transformation of Plasmids into Electro-competent Cells

0.66 μL of single plasmid in DH10B cells, or 0.3 μL of plasmid and 0.3 μL of pLysS in BL21(DE3), were added together on ice, flicked 5-6 times, and added into electro cuvette. Cuvettes were shocked and then grown in SOC (New England Biolabs) for 45-60 min at 37°C in incubator shaking at 250rpm. Culture was plated onto appropriate selection plates and colonies grown overnight at 37°C. For plasmid purification, several colonies were picked, cultured in LB ampicillin overnight, and DNA harvested (Qiagen, Hilden, Germany). The plasmid DNA was then sent for Sanger sequencing (Genewiz, Southplainfield, New Jersey) using sequencing primers (ST1).

Supplementary Method 3.

Production of Proheads from Plasmids

Co-transformed strains of BL21(DE3) (ST3) were grown in 5 mL LB amp^{50}/cam^{25} overnight. The culture was diluted 1:300 into TYM-5052 amp^{50}/cam^{25} in a Fernbach flask and grown for 24 hours at 37°C

shaking at 300 rpm in a shaker with 1-inch throw.[60] Cells were chilled for 30 min on ice. Samples were kept cold from this point forward unless explicitly stated. Cells were collected by centrifugation in JA-16.25 (Beckman Coulter, Brea, CA) at 6k rpm for 10 min and resuspended on ice in 60 mL Lysis buffer for Prohead 2 and D3 P1 Lysis buffer for Prohead 1. Resuspended cells were transferred to 250 mL flask, put on ice, and 1.2 mL 10% (w/v) Triton X-100 was added dropwise. While stirring, cells were cycled by heating to 23-24°C, holding for 5 min at temperature and chilling for 5 min on ice. 40 mL more of lysis buffer was added and cycled again. An additional 0.8 mL 10% Triton was added and cycled again. 1 mL of 10% Triton was added and cycled while heating to 27-28°C before holding. A further 50 mL of lysis buffer was added along with 1 mL 10% Triton. By this point, the cells would have lysed and be very viscous. After lysis, 2 mL of 1M $MgSO_4$ was added dropwise and 2 mL of 1 mg/mL DNase I was added. Lysed cells were heated to 28°C and held for 3 min or until viscosity dropped then chilled on ice for 5 min. Cell lysate was clarified by centrifugation in JA-16.25 at 8k rpm for 12 min, and PEG precipitated with 7% (w/v) PEG 8000 and 0.5 M salt (NaCl for P2, potassium glutamate for P1), and put on ice for 20 min. Precipitant was pelleted in JA-16.25 at 8k rpm for 16 min and resuspended in G buffer for P2 and D3 P1 buffer for P1. Resuspended pellet was further centrifuged in JA-16.25 at 8k rpm for 12 min to further remove any remaining insoluble material. Afterwards, the supernatant was centrifuged in Ti-45 (Beckman) at 35k rpm for 2 hr, and resultant pellet was covered with appropriate buffer (enough to cover pellet while tube stands upright). Pellet was allowed to resuspend slowly overnight. Resuspended pellet was clarified further to remove insoluble material in JA-18 (Beckman) at 8 k rpm for 10 min. This supernatant was loaded onto 10-30% glycerol gradients made with Gradient Master (Biocomp Instruments, New Brunswick, CA) in same buffer, and sedimented in SW-32Ti (Beckman) at 29k rpm for 105 min. The prohead band was extracted and dialyzed in dialysis buffer for P2 and D3 P1 dialysis buffer for P1 at room temperature. The dialyzed material was run over a POROS HQ20 anion exchange column using BIOCAD 700E perfusion chromatography system (Applied Biosystems, Waltham, MA). Collected peaks were pelleted in Ti-45 at 35k rpm for 2 hr and covered in appropriate buffer (enough buffer to cover the pellet while centrifuge tube is tilted on its side to maximize concentration). The pellet was allowed to resuspend slowly overnight.

Supplementary Method 4.

Small Scale Expression of Capsid Genes from Plasmids

Co-transformed strains of BL21(DE3) were grown in 2 mL minimal media MDG amp^{50}/cam^{25} overnight at 37°C shaking at 250 rpm with 2 inch throw.[60] 1.5 mL of TYM-5052 amp^{50}/cam^{25} in a 18x150 mm test tube was inoculated with 5 µL of MDG overnight and incubated at 37°C shaking at 250 rpm for 24 hr.[60] The cells were poured into 1.5 mL eppendorf tubes and harvested by centrifugation in a micro-centrifuge at 7krpm for 2 min (cold, if possible). The cells were resuspended in 300 µL of Lysis buffer for P2 or D3 P1 Lysis buffer for P1 by vortexing violently until fully resuspended. 4.5 µL of 10% Triton X-100 was added and the cells vortexed briefly and put on dry ice for 10 min. The cells were thawed slowly at room temperature for 15 min. 12 µL of 1M $MgSO_4$ and 9 µL of 1 mg/mL deoxyribonuclease I (in 1mM HCl) were added and mixed into the cells then heated at 30°C for 3 min or until viscosity dropped. Cell lysate was clarified by centrifugation at15k rpm for 10 min (cold, if possible). The insoluble fraction was resuspended with 300 µL G or D3 P1 buffer and saved for analysis. 50 µL of cell lysate was set aside for analysis. 250 µL of cell lysate was PEG precipitated with 250 µL 12% (w/v) PEG 8000/1M NaCl for P2 or 66 µL 3M K Glutamate and 79µL 30% (w/v) PEG 8000 for P1, and put on ice for 20 min. Precipitate was pelleted in micro-centrifuge at 15k rpm for 10 min in cold, and resuspended in 250 µL G or D3 P1 buffer for P2 and P1, respectively.

Supplementary Method 5.

Gel Electrophoresis

Native Agarose Gel Electrophoresis

0.36 g of LE agarose was melted in 30 mL of TAMg [40 mM Tris-HCl, 20 mM acetic acid pH 8.1, 1 mM $MgSO_4$] and poured into a mold. The gel was put into a submarine gel box (Hoeffer, Holliston, MA). 4 µL of sample was mixed 1 µL dye [50% (v/v) glycerol, 0.025% (w/v) bromophenol blue, 0.025% (w/v) xylene cyanol XFF] and 5 µL of TAMg. 4 µL was loaded onto the gel, and ran at ~120 V for 1 hr. The gels were stained with ~0.002% (w/v) Coomassie Brilliant Blue R250 in ~4.5% (v/v) methanol, ~9.9% acetic acid overnight, destained with 10% acetic acid, and photographed wet.

Sodium Dodecyl Sulfate Polyacrylamide Gel Electrophoresis

Samples were TCA (trichloroacetic acid) precipitated[67] by adding 25 µL 100% (w/v) TCA + 20 mg/mL deoxycholate[68] into diluted sample (10 µL sample + 225 µL 5 mM Tris-HCl pH 7.5), vortexing, and placing on ice for 10 min. Precipitate was pelleted in micro-centrifuge at max speed for 10 min. Pellet was suspended with 0.5 mL 90% (v/v), 10 mM Tris-HCl pH 7.5 at room temperature, and pelleted in micro-centrifuge at max speed. The supernatant was aspirated off, and the pellet dried under vacuum for 5 min. Pellet was suspended with 40 µL SDS sample buffer (62.5 mM Tris-HCl pJ 6.8, 2% (w/v) SDS, 10% (v/v) glycerol, 5% (v/v) Beta-mercaptoethanol) by vortexing, and heated in a boiling bath for 2.5 min. SDS samples were loaded and run on 12% SDS polyacrylamide minigel using original buffers described by Laemmli.[65] A low-crosslinker recipe (33.5% (w/v) acrylamide + 0.3% (w/v) methylene bisacrylamide)[66] was used. Gels were stained with 0.5% (w/v) Coomassie Brilliant Blue R250 in 50% (v/v) Methanol and 10% acetic acid, destained with 10% acetic acid, and photographed wet.

Supplementary Tables

Primer Name	Sequence
D3right1	CGCAAGCTTGCCGGTCTCCCGGCCCCT
D3left1	CGGCTCGAGCTCATGAACTGGTTCAGCCAGCCA
D3seq1	GCCGTCGACAAAGGGTGGGCA
D3seq2	CAGGTAAACACCCAAATCGCC
D3seq3	AGTCGCTCATTGCGATTCTCC
d3p1	AATGGCCAGGCCTTCGAATACGTCGC
d3p2	GGCTCGGCTGGGCGCTCATTTC
d3p2a	AGTAGGATCCGGCTCGGCTGGGCGCTCATTTC
D3p-33	CGGCTCGAGCCACCCCCGCCAGCAGCGCCA
D3portN	GAGTGTACGAGCGGAAAGC
D3portC+SphI	TAGTACGCATGCCGCTGCCGGAAGATTT
DP135	GATCATCAGGAAGGCGGCGCGGC
DP136Q	CAGAACGCCTGGCTGATCGCCAT
T7phi10	TCACCGTCATCACCGAAACGC
RD96-55	TCGCTGAGATAGGTGCCTC
D3seq1	GCCGTCGACAAAGGGTGGGCA
D3seq3	AGTCGCTCATTGCGATTCTCC

Table S1.

Primers used for this study. Sequences are given in 5' to 3' direction. pT7-5 sequence in black; D3 sequence in blue; Mutant sequence in red. T7phi10, RD96-55, d3seq1, and d3seq3 were sequencing primers. Primers were purchased from Integrated DNA Technologies (Coralville, IA).

Name	Description	Notes	Reference
pLysS		*p15Aori, T7 lysozyme, [*Cm^R*].*	Studier 1991[69]
pT7-5		*T7 promoter[*Amp^R*].*	Vos 1988[70]
pD3	pT7-5::D3gene5gene6	Primers *D3right1* and *D3left1* used in amplification of phage D3 genome insert. Digested with *XhoI* and *HindIII* and subcloned into pT7-5 vector, which was digested with SalI and HindIII.	This study
pD3Δ1	pT7-5::D3gene6	*ApoI* and *EcoRI* digest of *pD3* to delete protease gene. Ligated *ApoI->EcoRI* piece to itself.	This study
pD3i	pT7-5::D3gene5H141Qgene6	*DP135* and *DP136Q* primers used for site mutagenesis with *D3right1(rev)* and *D3left1* in amplification of *pD3* insert with H141Q mutation of protease gene. Digested with *XbaI* and *SphI* and subcloned into *pD3* vector.	This study
pD3P	pT7-5::D3gene4gene5gene6	Primers *d3p1* and *d3p2a* used with phage D3 genome to amplify insert. Digested with *BamHI* and *NsiI*, and subcloned into *pD3* vector.	This study
pD3Δ2P	pT7-5::gene4gene6	*pD3P* digested with *EcoRI*. Ends filled with Klenow polymerase (Boehringer Mannheim, Ingelheim, Germany) and dNTPs and religated. Insert was amplified with primers *D3portN* and *D3portC+SphI* from *pD3P* Klenow filled plasmid (*pD3PK*). Digest insert with *ApoI* and *BamHI* and subclone into *pD3PK* vector.	This study
pD3iP	pT7-5::gene4gene5H141Qgene6	*DP135* and *DP136Q* primers used for site mutagenesis with *D3right1(rev)* and *D3left1*in amplification of *pD3* insert with H141Q mutation of protease gene. Digested with *XbaI* and *SphI* and subcloned into *pD3P* vector.	This study

Table S2.

Plasmids used for this study.

Plasmid Name	Description	Reference
**For Plasmid Purification**		
	DH10B	Durfee 2008[71]
**For Protein Expression**		
	BL21(DE3)	Studier 1986[72]
pD3	BL21(DE3) pLysS pD3	This study
pD3Δ1	BL21(DE3) pLysS pD3Δ1	This study
pD3i	BL21(DE3) pLysS pD3i	This study
pD3P	BL21(DE3) pLysS pD3P	This study
pD3Δ2P	BL21(DE3) pLysS pD3Δ2P	This study
pD3iP	BL21(DE3) pLysS pD3iP	This study

Table S3.

Bacterial strains used for in this study.

D3 Portal Gene

GTGAGTAAGAGTCTCGGAAAAGTCCTGAGCAGTGCTACGTCTGCGCCCAGGTCTTCATTGTTCGGCTGGGGGGGTAAGACCATCCGCCTGA
CAGATGGCGCGTTCTGGTCGCAGTTCCTGGGGCGAGAGTCGTCTAGCGGAAAAAAAGTCACTGTCGACAAGGCAATGAAGCTGTCTGCGGT
ATGGGCTTGTGTTCGCTTGATCTCTACTTCTGTCGCCGGTCTTCCGCTTGGAGTGTACGAGCGGAAAGCGGACGGAAGCAGAGTCGATGCT
CGGTCGTTCCCGCTCTACGATGTTGTTCACAACAGCCCCAACGACGACATGACGGCCTTCCAGTTCTGGCAGGCCATGGTCGCATCGATGC
TGCTTTGGGGTAACGCATACGCGGAGATTCGCCGCGCTGCGGGCAGACCGGCTGCGTTGGACTTCCTGCTTCCATCGAGGGTCGACCTGGA
GTGTGATGAAAACGGTCGGCTGAAGTACTTCTATACGACAAAGAAGGGTGCTCGTAGAGAGATCGAGCGTACTAACATGCTGCACATCCCG
GCGTTCACGCTGGATGGTCGAATTGGTCTCTCTGCAATCAGATACGGCGTTGATGTCTTCGGCTCGGTCATGTCGGCGGAGGACGCAGCCA
ACGGCACATTCAAGAACGGACTTCTACCCACGGTCGCATTCAAGGTTGATCGCATTCTCCAGCCTGCGCAGCGGGAGGAGTTCAGGGAGTA
TGTGAAGTCCGTATCGGGCGCGATGAACTCCGGAAGATCCCCGGTTCTGGAGCAGGGGATTACCCCTGAAACCATCGGCATCAATCCGGTC
GATGCTCAGTTGCTGGAGACGCGAGAGCATGGCGTGATCGAGATTTGCAGATGGTTCGGGGTGCCGCCCTGGATGATCGGCCAGACCGACA
AGGGGAGCAACTGGGGGACAGGGCTTGAACAGCAGATGCTCGCGTTCCTGACATTCTCGATCAGCTCGATCACCAATCAGATTCAGCAGTG
CGTCAACAAGCGGCTGCTAACTGCGCCCGAGCGGATTCGCTATTACGCCGAGTTCTCACTTGAGGGGTTCCTGAAGGCTGATAGCGCTGGT
CGCGCTGCCTGGTACAGCACTATGGCGCAAAACGGTTTCATGACCCGCAACGAAGGTCGCCGGAAAGAGAACCTGCCAGAACTCCCCGGCG
GAGACATTCTCACCGTCCAATCCAACCTAGTTCCAATCGACCAACTCGGTCAATCTAACGAGAGCCAGGCCGTCCGCGCCGCGCTCATGAA
CTGGCTCAGCCAGCCAGAACCACAGGAGTAA

D3 Protease Gene

ATGACTCTGCGAAATCTTCCGGCAGCGCCGGAGGCTCGCCCGCGCTCGGGCGTCCAGTGCGACCTGGCGCCCAAAGCGCTAGATGCATGGC
GTCCTGAGCTTCGAGCAGCTTCTGGCGATAACCCGGACTCCACGATCACCATCTACGAGCCGATTGGCTACGACTGGTGGACCGGTGAAGG
TGTCACGGCAAAACGCATTGCTGGCGCTCTGCGCTCCATCGGCAACGATGTCGATGTGACCGTGAACATCAACAGCCCCGGCGGCGACGTA
TTCGAAGGCCTGGCCATTTACAACCTGCTGCGCGAGCACAAGGGCAAGGTCACGGTGAACATCATCGGCCTGGCTGCCTCTGCCGCCTCTT
TCATCGCCATGGCGGGGGATGAAATCCGCATCGGCCGCGCCGCCTTCCTGATGATCCATAACGCCTGGCTGATCGCCATGGGTAATCGGAA
TGATCTCCGTGAGATAGCCGATTGGCTGGAGCCATTCGACATGACGCTGGCTGACATTTACGCACAGCGCACGGAAATCGACATCGACGAC
ATCGTGAAACAGATGGACGCCGAGACCTGGATCGGCGGGCGCGAAGCCGTCGACAAAGGGTGGGCAGATGCCTTCCTGGAGTCCGACGAGA
TCTCCAGCGCTCCCAGCAACCGCAGCGAAGCCATCCTGGCCAAGCGCCGGATGGATGCCGCCCTGGCTCGCAGCGGAATGCCGCGAAGCCA
GCGCAATGAACTCATCAACGACTTCAAGACCAGCATGCTTGGCGCTGCTGGCGGGGGTGGTGACACCCCGACCGATATGCCTGGCGCTGTC
GCTCCTGACCTCTCCGCTGCACTACGGGCAGCACAAGACATCACCAAATTCCTCCAAGGAGAATCGCAATGA

D3 Major Capsid Gene

ATGAGCGACTTCGAAAAACAAATCGGCGAACTGAACGCCAGCCTCAAGCAGGTCGGGGACCAAATCAAGTCCCAGGCCGAACAGGTAAACA
CCCAAATCGCCAATTTCGGCGAAATGAACAAGGAAACCCGCGCCAAGGTCGACGAACTGCTGACTGCCCAGGGCGAACTGCAAGCACGACT
GAGCGCCGCGGAACAAGCCATGCTGGCCAACGAGAAGCGTGACGGCGGCGAGGAAGCACCGAAGACCGCCGGCCAAATGGTCGCAGAGAGC
CTGAAAGAGCAGGGTGTAACCAGCTCCCTGCGCGGCTCGCATCGCGTATCCATGCCGCGCTCGGCCATCACCTCCATCGACGGCTCTGGCG
GCGCCCTGGTGGCTCCTGATCGTCGCCCCGGTGTCGTTGCCGCTCCGCAGCGTCGACTGACCATCCGCGACCTGGTTGCGCCTGGCACCAC
TGAGTCGAACTCCGTCGAGTACGTCCGCGAGACCGGCTTCGTCAACAATGCCGCTCCTGTTTCGGAAGGCACCCAGAAGCCATACTCCGAC
CTGACCTTCGAACTGGAAAACGCGCCGGTTCGCACCATCGCACACCTGTTCAAGGCAAGTCGCCAGATCCTGGACGACGCTTCGGCCTTGC
AGAGCTACATCGATGCGCGCGCTCGTTACGGCCTGATGCTGGTCGAAGAAGGTCAACTGCTCTACGGGAACGGGACCGGCGCCAATCTGCA
CGGCATCATTCCGCAGGCACAGGCCTACGCGCCGCCGAGTGGCGTAGTGGTAACCGCCGAGCAGCGAATCGACCGCATCCGCCTGGCGATC
CTTCAGGCGCAACTGGCCGAGTTCCCGGCCAGCGGTATCGTGCTCAACCCCATCGACTGGGCGCTGATCGAGCTGACCAAGGACGCCGAGA
ACCGTTACATCATCGGCAGCCCGCAGAACGGCACCACTCCGACCCTCTGGCGTCTGCCGGTGGTGGAAACCCAGGCCATCACTCAGGACGA
GTTCCTGACCGGTGCGTTCTCTCTCGGCGCCCAGATCTTCGACCGCATGGACATCGAGGTTCTGGTTTCCACCGAGAACGACAAGGACTTC
GAGAACAACATGGTCACCATCCGCGCTGAGGAGCGGCTGGCCTTCGCGGTCTATCGCCCCGAGGCTTTCGTGACTGGTTCGCTGACCGCCA
GCTAA

Table S4.

Gene Sequences. Notes:

- Sanger sequencing of plasmids containing D3 genes showed discrepancies compared with reported sequence of the D3 genome (Genbank: AF165214) by Kropinski.[73] Shown is corrected sequence per Sanger sequencing results with discrepancies highlighted in red.
- 6 errors in the published D3 sequence have been confirmed and led to nucleotide changes in the coding sequences. They are (erroneous nucleotide in red):
  - AAG->GAG (3506) and TTC->CTC (3542) in gene 4
  - ATT->ACT (3576) and GTT->GCT (3606) in gene 5
  - TGT->GGT (5106) and AAC->ACC (5354) in gene 6.
- Our revised genome sequence was deposited to Genbank (PV029250).

**D3 Portal Protein**<br>MSKSLGKVLSSATSAPRSSLFGWGGKTIRLTDGAFWSQFLGRESSSGKKVTVDKAMKLSAVWACVRLISTSVAGLPLGVYER<br>KADGSRVDARSFPLYDVVHNSPNDDMTAFQFWQAMVASMLLWGNAYAEIRRAAGRPAALDFLLPSRVDLECDENGRLKYFYT<br>TKKGARREIERTNMLHIPAFTLDGRIGLSAIRYGVDVFGSVMSAEDAANGTFKNGLLPTVAFKVDRILQPAQREEFREYVKS<br>VSGAMNSGRSPVLEQGITPETIGINPVDAQLLETREHGVIEICRWFGVPPWMIGQTDKGSNWGTGLEQQMLAFLTFSISSIT<br>NQIQQCVNKRLLTAPERIRYYAEFSLEGFLKADSAGRAAWYSTMAQNGFMTRNEGRRKENLPELPGGDILTVQSNLVPIDQL<br>GQSNESQAVRAALMNWLSQPEPQE
**D3 Protease Protein**<br>MTLRNLPAAPEARPRSGVQCDLAPKALDAWRPELRAASGDNPDSTITIYEPIGYDWWTGEGVTAKRIAGALRSIGNDVDVTV<br>NINSPGGDVFEGLAIYNLLREHKGKVTVNIIGLAASAASFIAMAGDEIRIGRAAFLMIHNAWLIAMGNRNDLREIADWLEPF<br>DMTLADIYAQRTEIDIDDIVKQMDAETWIGGREAVDKGWADAFLESDEISSAPSNRSEAILAKRRMDAALARSGMPRSQRNE<br>LINDFKTSMLGAAGGGGDTPTDMPGAVAPDLSAALRAAQDITKFLQGESQ
**D3 Major Capsid Protein**<br>MSDFEKQIGELNASLKQVGDQIKSQAEQVNTQIANFGEMNKETRAKVDELLTAQGELQARLSAAEQAMLANEKRDGGEEAPK<br>TAGQMVAESLKEQGVTSSLRGSHRVSMPRSAITSIDGSGGALVAPDRRPGVVAAPQRRLTIRDLVAPGTTESNSVEYVRETG<br>FVNNAAPVSEGTQKPYSDLTFELENAPVRTIAHLFKASRQILDDASALQSYIDARARYGLMLVEEGQLLYGNGTGANLHGII<br>PQAQAYAPPSGVVVTAEQRIDRIRLAILQAQLAEFPASGIVLNPIDWALIELTKDAENRYIIGSPQNGTTPTLWRLPVVETQ<br>AITQDEFLTGAFSLGAQIFDRMDIEVLVSTENDKDFENNMVTIRAEERLAFAVYRPEAFVTGSLTAS

Table S5.

Protein Sequences. Notes:

- 6 errors in the published D3 sequence have been confirmed and led to amino acid changes in the coding sequences. They are (erroneous nucleotide in red):
  - K415E and F427L in gene 4
  - I2T and V12A in gene 5
  - C230G and N299T in gene 6

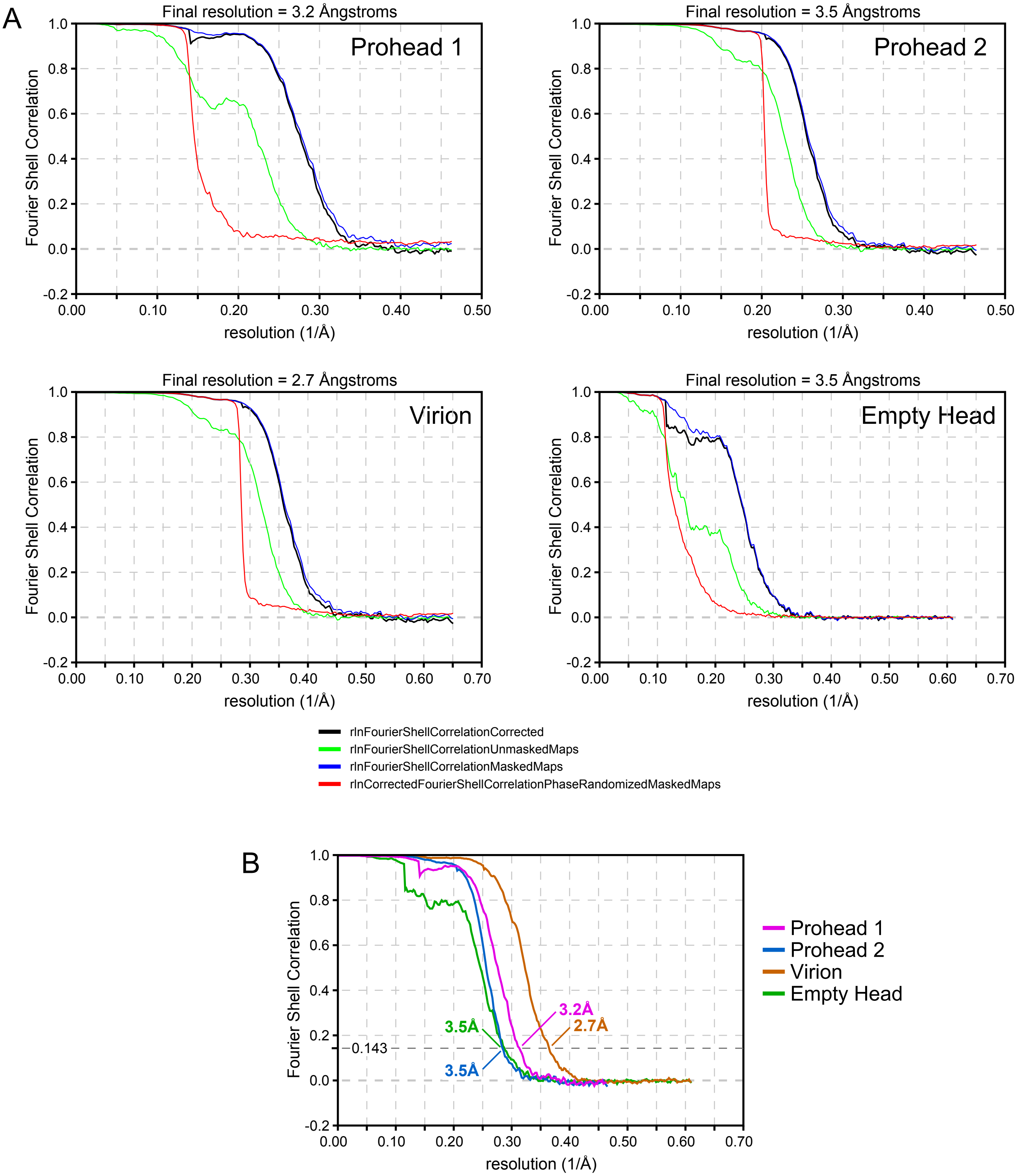

Supplementary Figure S1. (A) Fourier Shell Correlation (FSC) curves for estimating resolution of each density map, as reported by Relion. (B) The *rlnFourierShellCorrelationMaskedMaps* curves for all density maps, including resolution estimates from where the curves cross the so-called "gold-standard" correlation coefficient limit of 0.143.

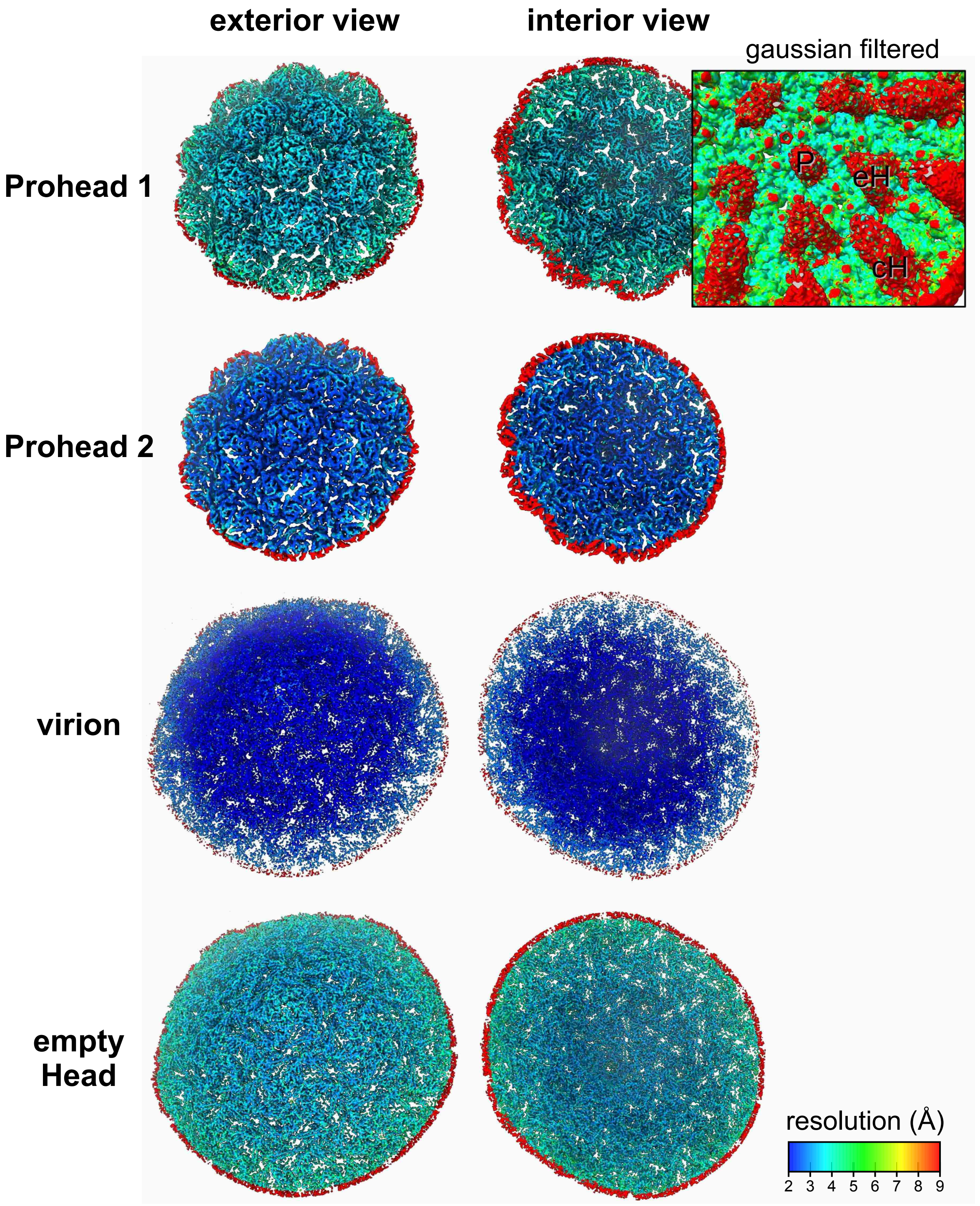

Supplementary Figure S2. Local resolution models of the D3 vertex density maps. The coiled-coil sub-domain density under the Prohead 1capsomers is only visible with gaussian filtering due to their flexibility, and with the contour level reduced to expose lower resolution density.

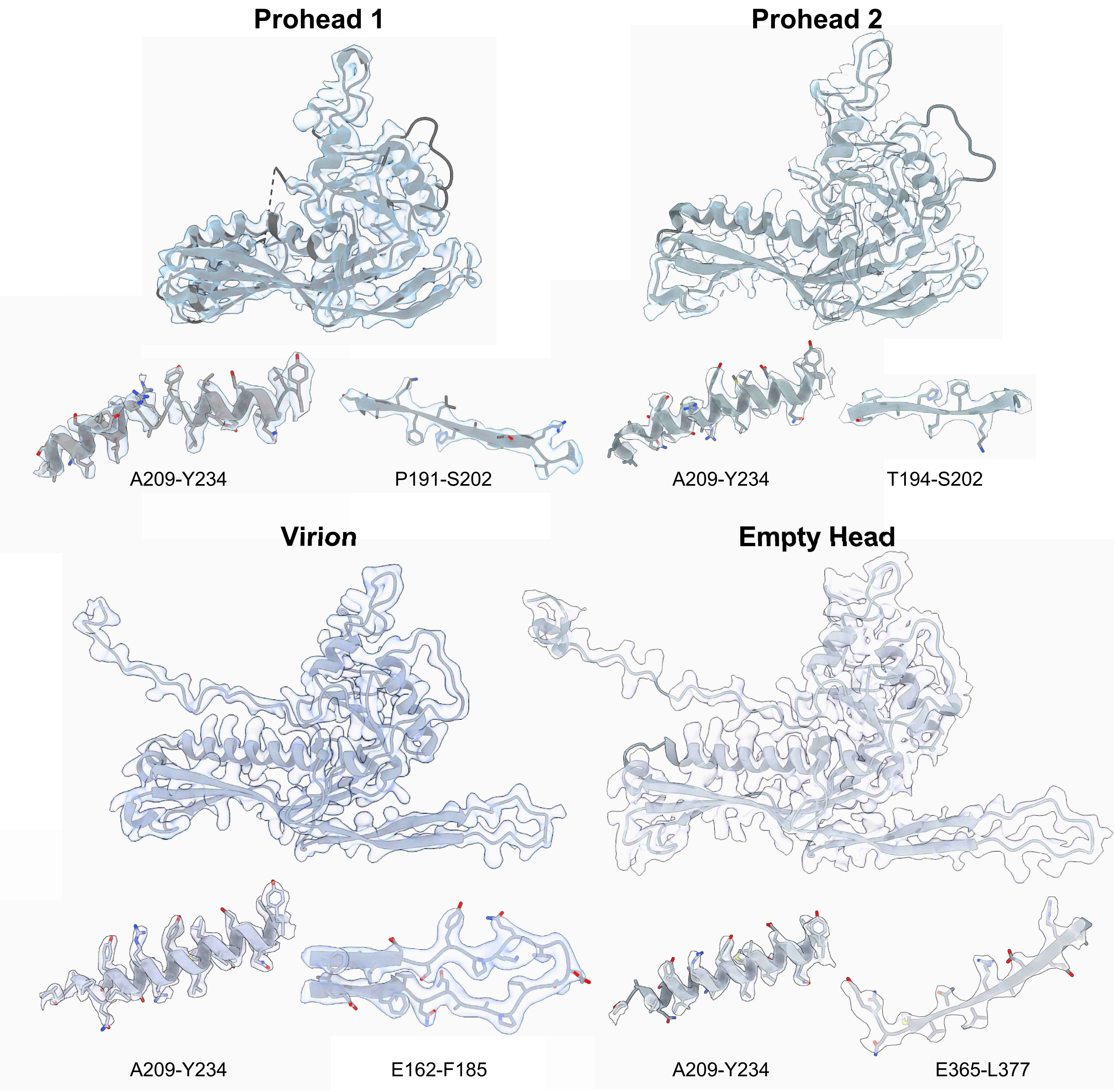

Supplementary Figure S3. Atomic models of the D3 MCP derived from the four capsid forms, as indicated, including representative regions of density withsidechain fits.

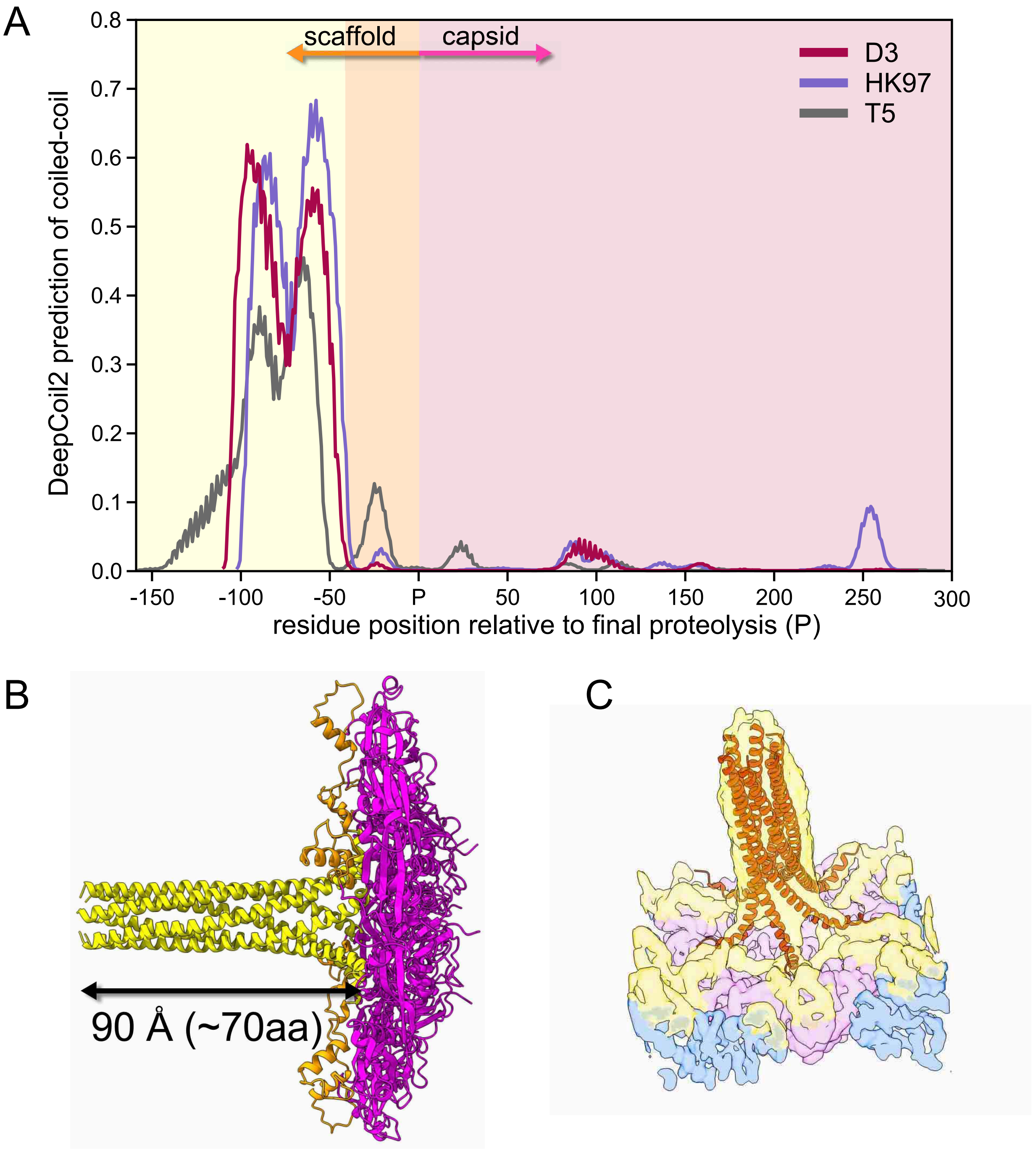

Supplementary Figure S4. (A) Propensity for the MCPs of three HK97-like phages to adopt a coiled-coil fold, as predicted from the amino acid sequences by DeepCoil2.[74] The N-terminal regions of the scaffold domains are strongly indicated to form coiled-coils, and the lengths correlate with capsid size: HK97 – scaffold domain of 101 residues, T=7; D3 – 111 residues, T=9; and T5 – 160 residues, T=13. (B) A capsomer model of five D3 MCP chains as predicted by Alphafold 2.[75] This predicted structure shows an extended coiled-coil fold (yellow) for the N-terminal region of the scaffold domain as well as a PDAR-like fold (orange) for the C-terminal part. The length of the coiled-coil fold (90Å) is compatible with the height of the towers of density that we observe in our Prohead 1 map. (C) Fit of coiled-coil sub-domains into the tower density under the center hexon of the Prohead 1 density map, demonstrating consistency of the model with the density.

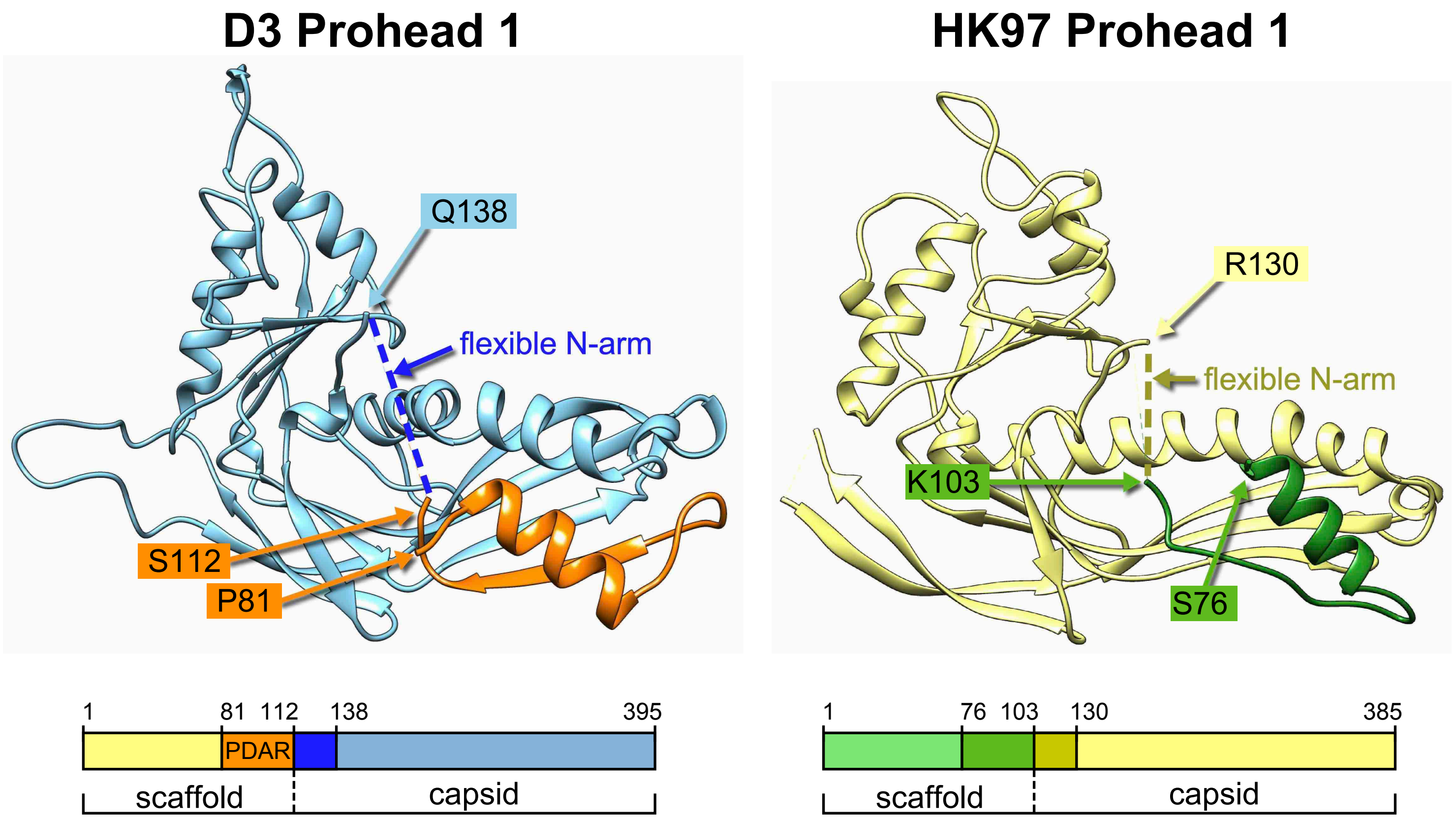

Supplementary Figure S5. Comparison of the Prohead 1 subunit models of phages D3 and HK97 indicating the boundaries of regions that could be modeled confidently from cryoEM reconstructions. In both cases, the N-arm of the capsid domains was unresolved, but the C-terminal portion of the scaffold was well resolved, including the PDAR (D3: orange, HK97: dark green). Data for HK97 are from Huet *et al*, 2023.[11]

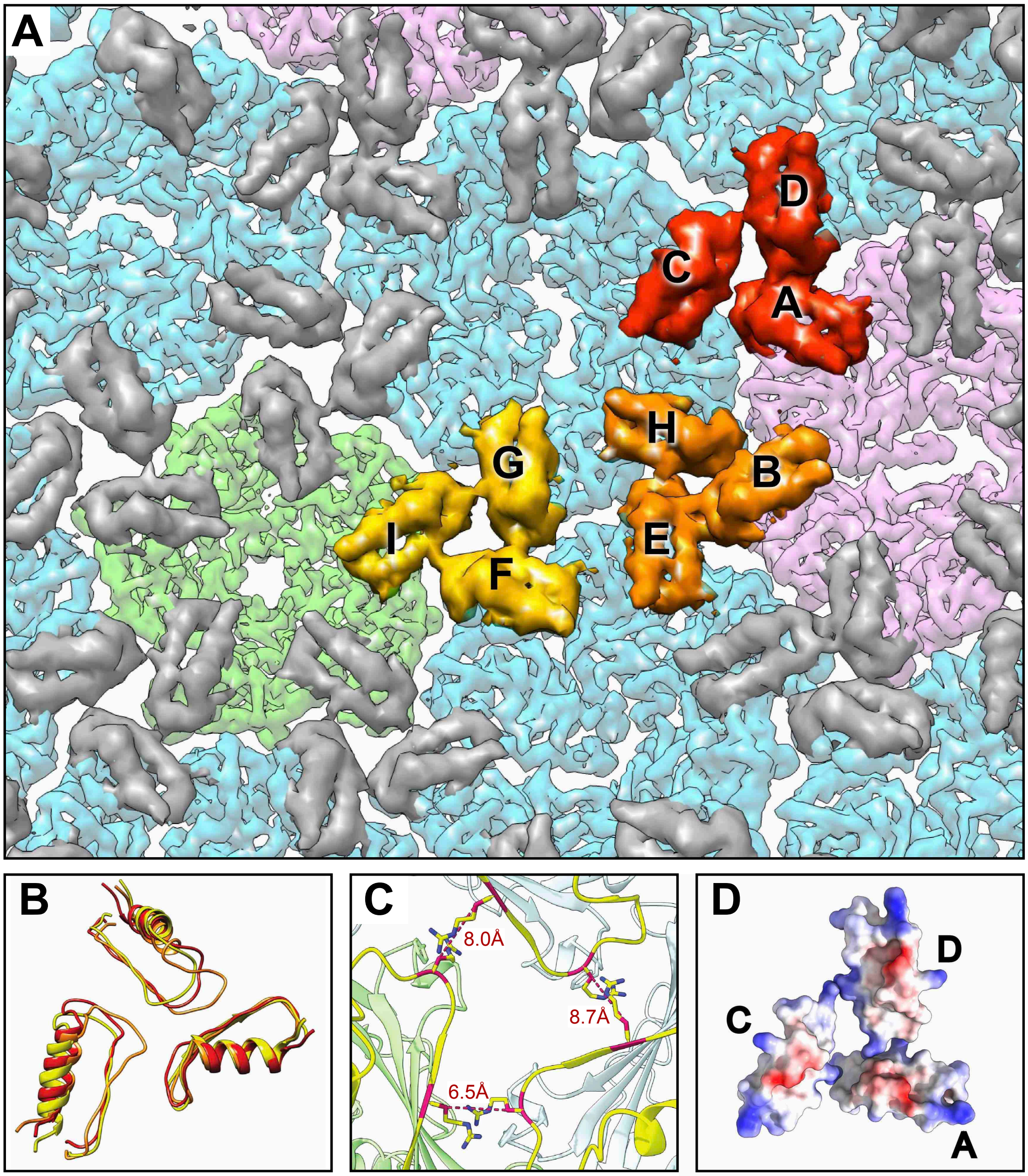

Supplementary Figure S6. (A) Low resolution density map of Prohead 1 highlighting the three unique PDAR trimers at each pseudo-3-fold location (B) The three PDAR timers overlaid, exhibiting orientation differences. (C) Distance measure between the β -carbon of the interacting arginine residues on participating subunits (I,G,F) of the PDAR trimer at the penton-edge hexon interface. (D) Electrostatic rendition of the PDAR surface demonstrating an absence of attractive interactions between the three subunits.

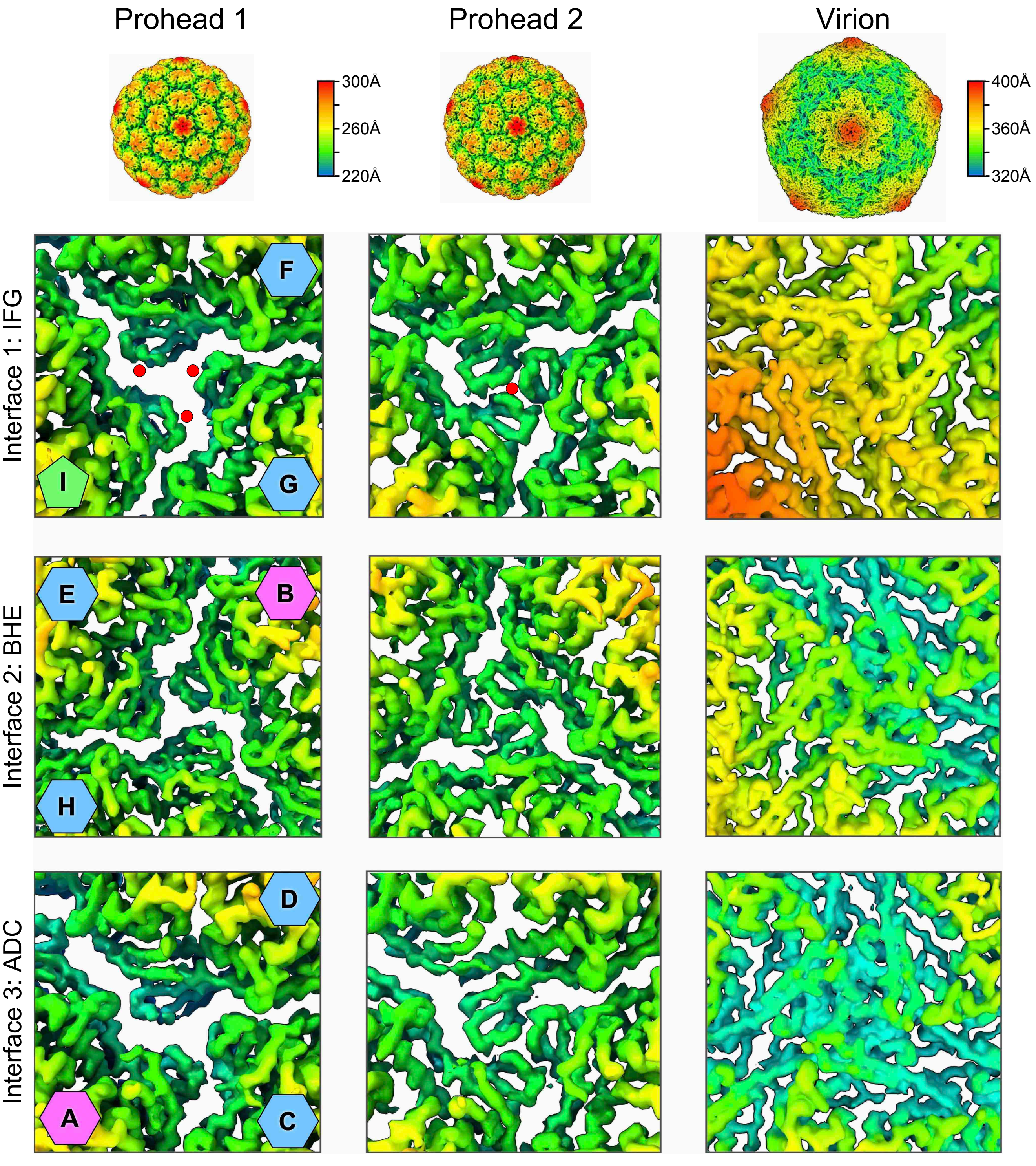

Supplementary Figure S7. Comparison of density distributions at the three local 3-fold sites, as indicated at left. Surfaces are colored by radius measured from the capsid centers, as shown by the scales. Note that none of the local 3-fold sites exhibits strict 3-fold icosahedral symmetry, thus subunit interactions are not identical within or between sites. Subunits are indicated by letter, and the source capsomer by the colored penton (green) andhexons (eH: blue, cH: pink), as shown in Figure 1C. Red dots in the Interface 1 row indicate P-loops that are in the “up” orientation (Prohead 1) and “down” orientation (Prohead 2), and the corresponding organizations are readily visible for Interfaces 2 and 3.

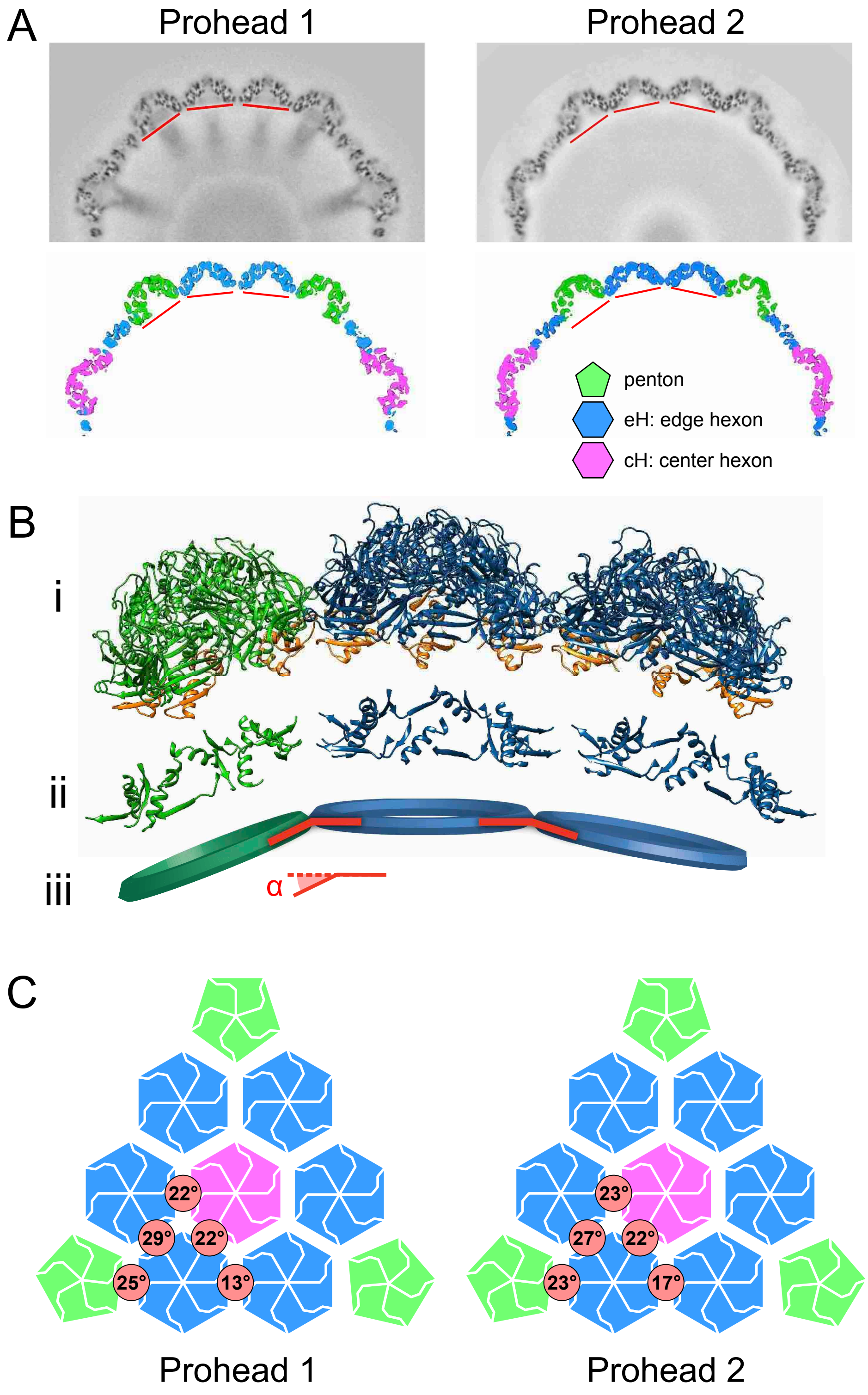

Supplementary Figure S8. (A) Central sections through the Prohead 1 and Prohead 2 density maps in grey scale (top) and color-coded according to capsomer, as indicated (bottom). The red lines indicate planes through each capsomer that are defined by residues 148-154, 207-222, and 348-358, as shown in the model view (B) for Prohead 1: (i)capsomers, including PDAR domains in orange; (ii) residues defining the capsomer planes; and (iii) a schematic of how dihedral angles between capsomers are measured. Larger angles indicate greater curvature. (C) Dihedral angles measured between capsomers, as given in the red circles.

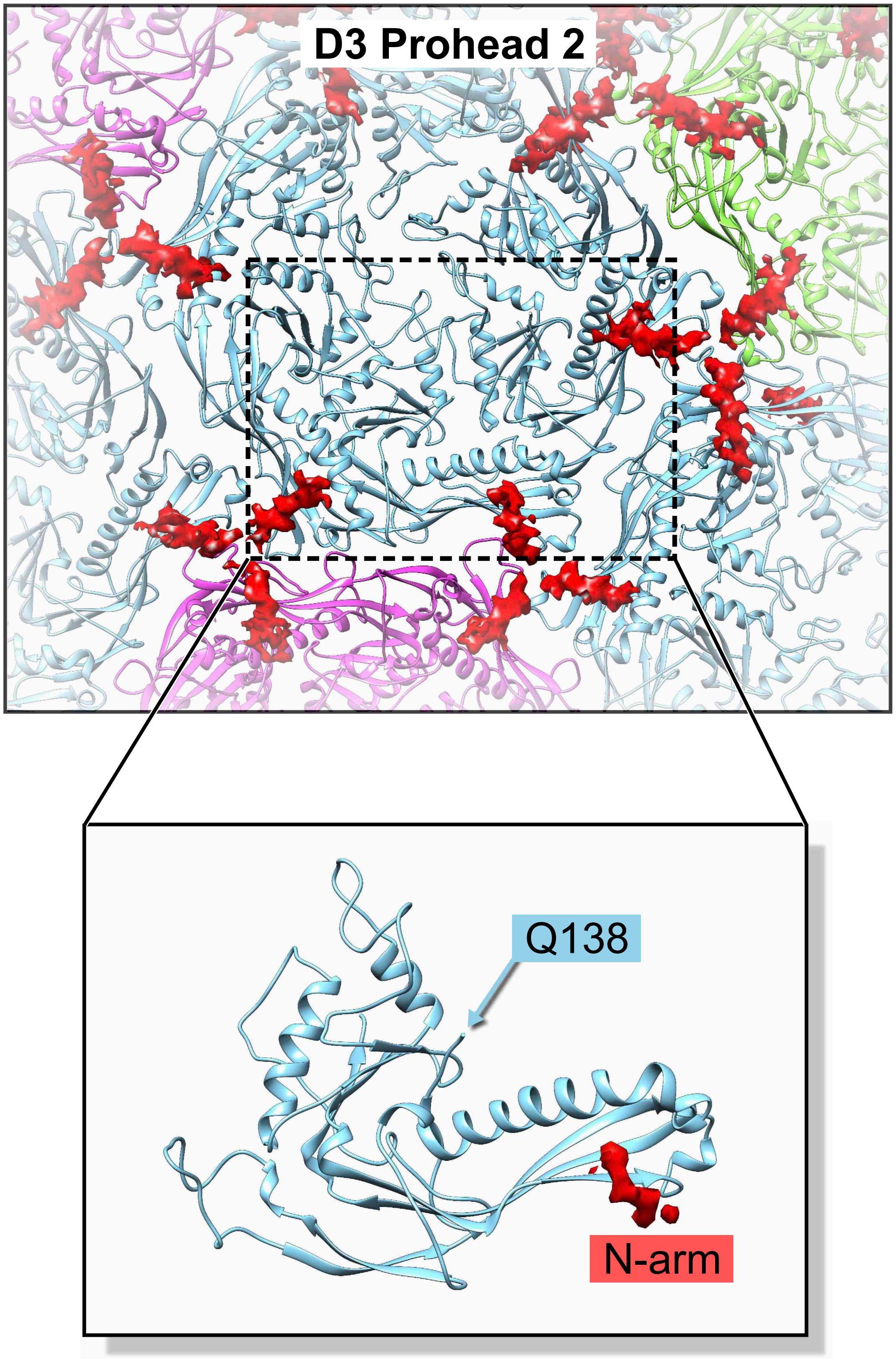

Supplementary Figure S9. Unmodeled density (red) in the D3 Prohead 2 map is consistent with the location of the amino-terminal part of the N-arm based on its location near the point of scaffold cleavage.

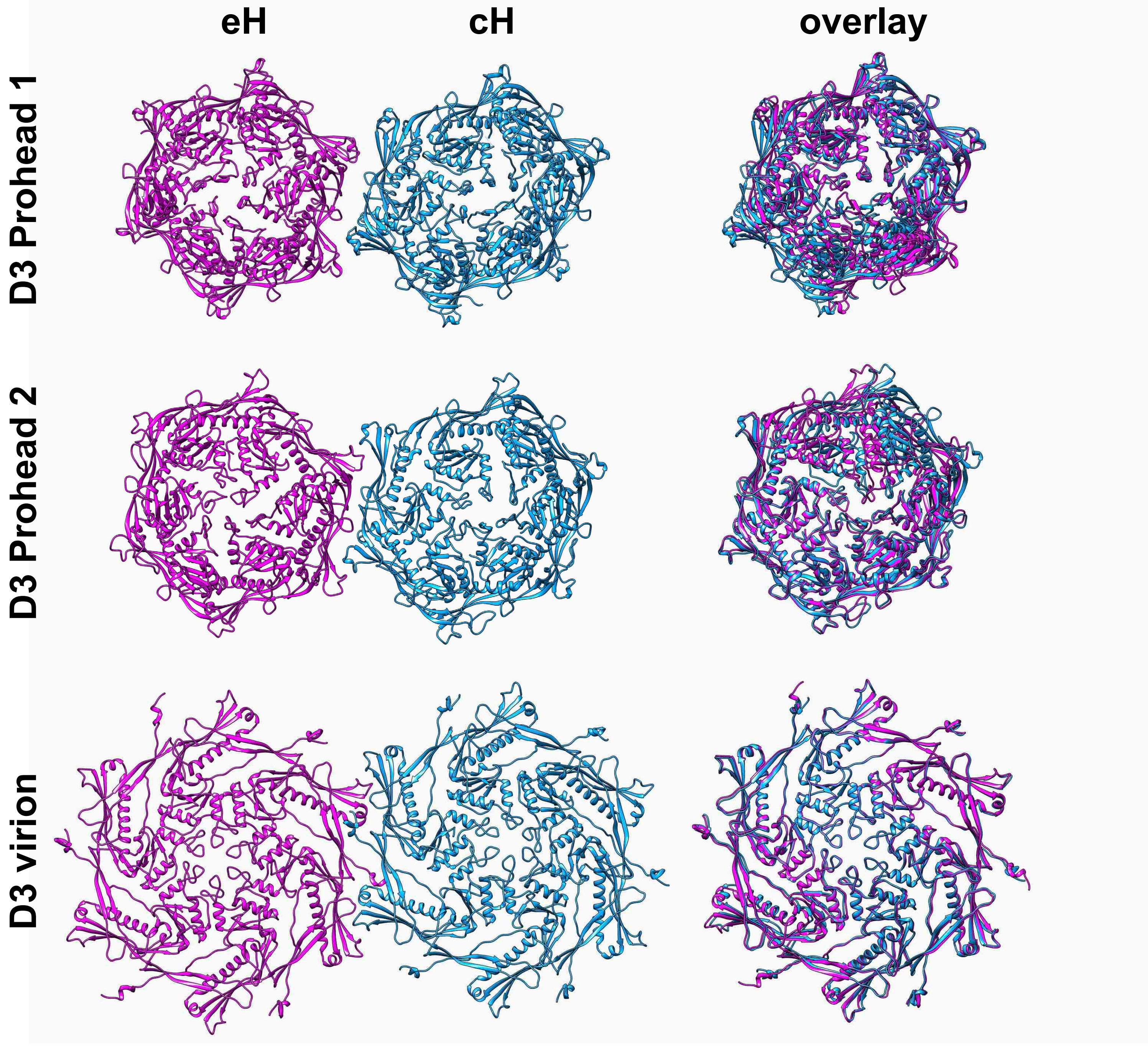

Supplementary Figure S10. Comparison of models for the edge hexon (eH) and center hexon (cH) in the various capsid forms. The virion and empty Head models appear virtually identical viewed in overlay so only the virion is shown here.

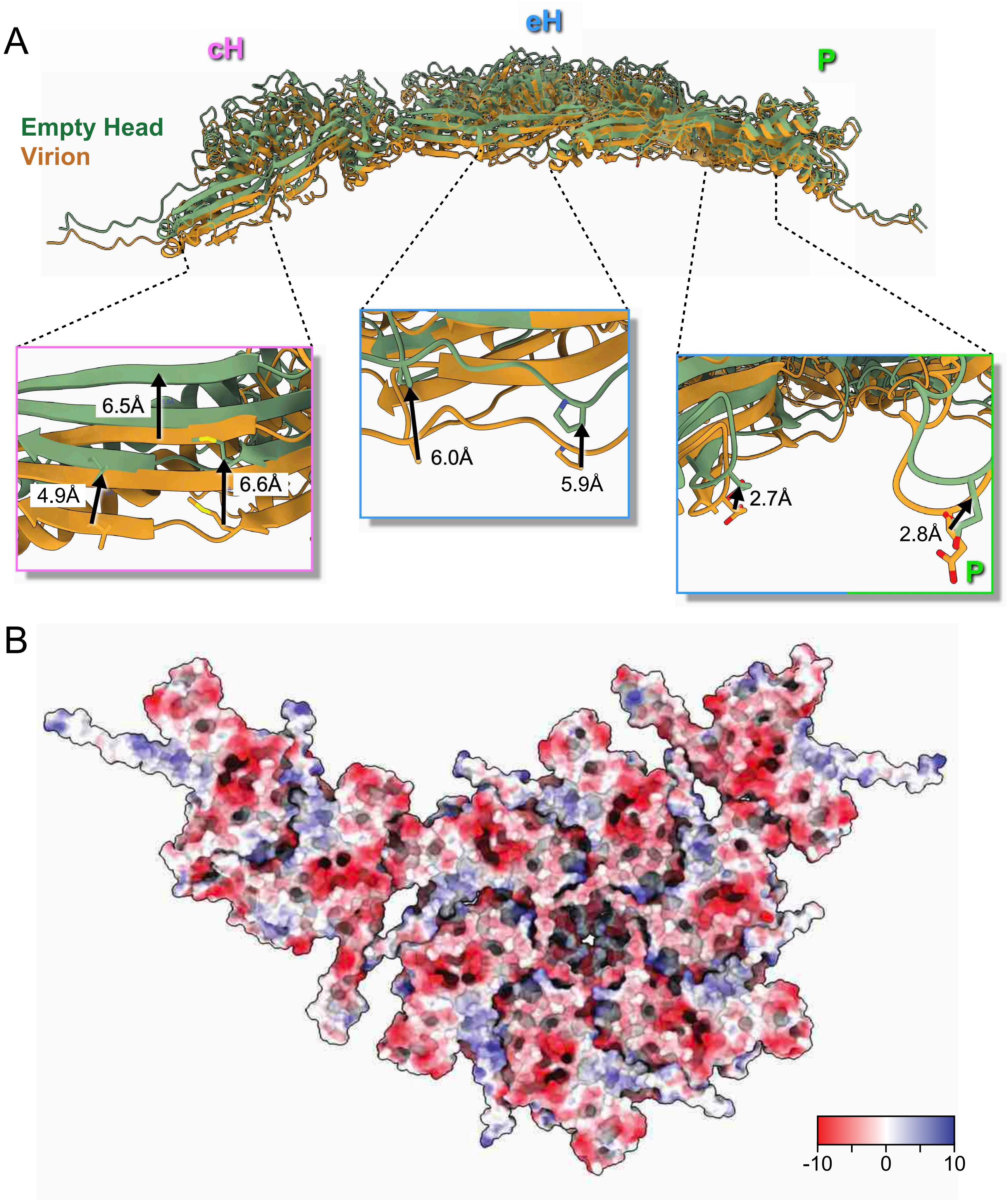

Supplementary Figure S11. (A) ASU of the Virion Head and empty Head, post-DNA ejection, demonstrating the range (~2-7Å) of radius difference between the capsid with and without packaged DNA. (B) Electrostatic potential of thevirion ASU viewed from the capsid interior.

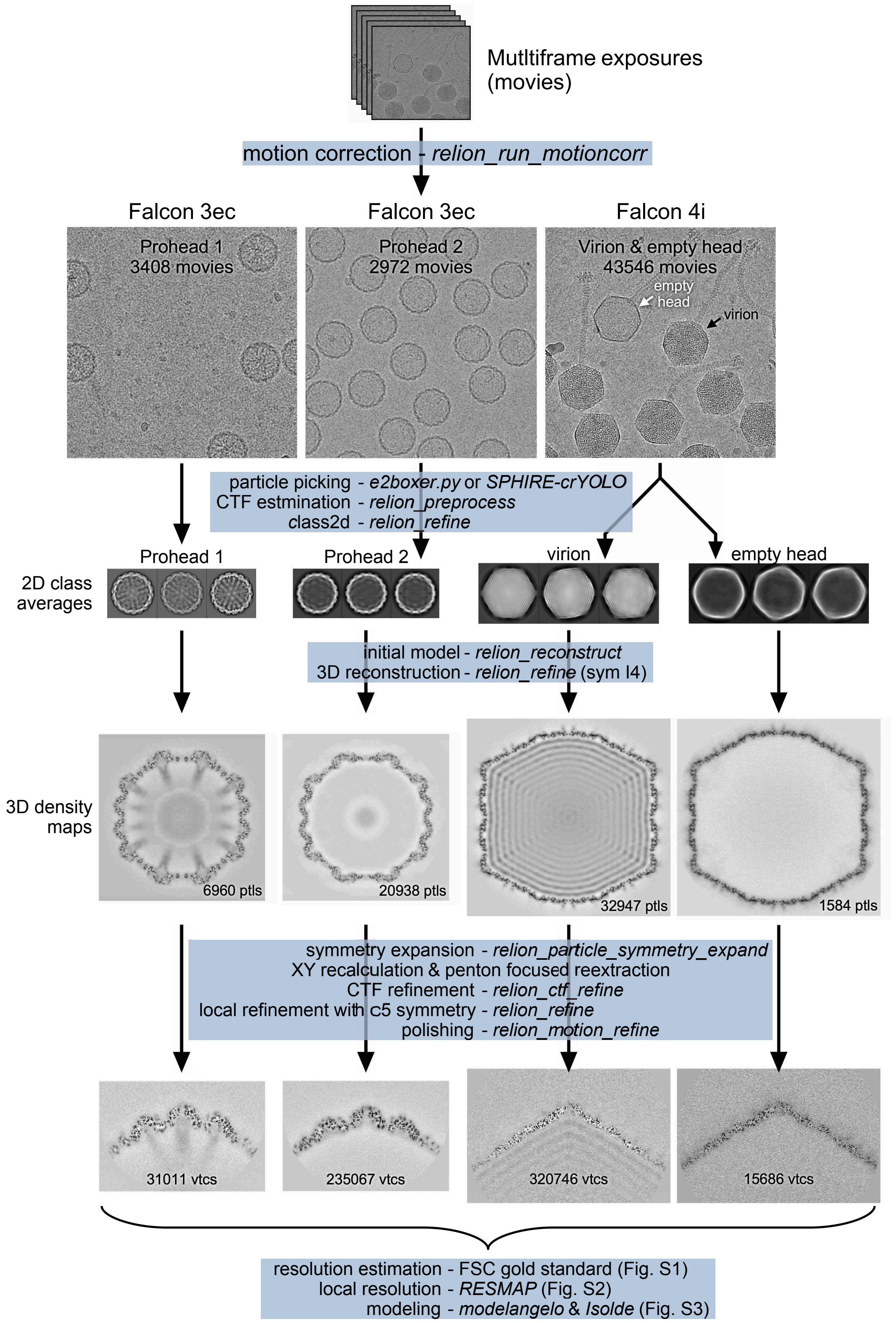

Supplementary Figure S12. Cryo-EM data processing workflow. “ptls” are counts of capsid particles; “vtcs” are counts of vertices.

62. Tabor S, Richardson CC. A bacteriophage T7 RNA polymerase/promoter system for controlled exclusive expression of specific genes. *Proc Natl Acad Sci U S A* **82**, 1074-1078 (1985).

63. Adereth Y, Champion KJ, Hsu T, Dammai V. Site-directed mutagenesis using Pfu DNA polymerase and T4 DNA ligase. *BioTechniques* **38**, 864, 866, 868 (2005).

64. Maurer JB, Oh B, Moyer CL, Duda RL. Capsids and Portals Influence Each Other's Conformation During Assembly and Maturation. *J Mol Biol* **432**, 2015-2029 (2020).

65. Laemmli UK, Molbert E, Showe M, Kellenberger E. Form-determining function of the genes required for the assembly of the head of bacteriophage T4. *J Mol Biol* **49**, 99-113 (1970).

66. Dreyfuss G, Adam SA, Choi YD. Physical change in cytoplasmic messenger ribonucleoproteins in cells treated with inhibitors of mRNA transcription. *Mol Cell Biol* **4**, 415-423 (1984).

67. Duda RL, Hempel J, Michel H, Shabanowitz J, Hunt D, Hendrix RW. Structural transitions during bacteriophage HK97 head assembly. *J Mol Biol* **247**, 618-635 (1995).

68. Maloy SR, Stewart VJ, Taylor RK. *Genetic analysis of pathogenic bacteria: A laboratory manual*. Cold Spring Harbor Laboratory Press (1996).

69. Studier FW. Use of bacteriophage T7 lysozyme to improve an inducible T7 expression system. *J Mol Biol* **219**, 37-44 (1991).

70. Vos P, *et al.* Infectious RNA transcripts derived from full-length DNA copies of the genomic RNAs of cowpea mosaic virus. *Virology* **165**, 33-41 (1988).

71. Durfee T, *et al.* The complete genome sequence of Escherichia coli DH10B: insights into the biology of a laboratory workhorse. *J Bacteriol* **190**, 2597-2606 (2008).

72. Studier FW, Moffatt BA. Use of bacteriophage T7 RNA polymerase to direct selective high-level expression of cloned genes. *J Mol Biol* **189**, 113-130 (1986).

73. Kropinski AM. Sequence of the genome of the temperate, serotype-converting, Pseudomonas aeruginosa bacteriophage D3. *J Bacteriol* **182**, 6066-6074 (2000).

74. Zimmermann L*, et al.* A Completely Reimplemented MPI Bioinformatics Toolkit with a New HHpred Server at its Core. *J Mol Biol* **430**, 2237-2243 (2018).

75. Jumper J*, et al.* Highly accurate protein structure prediction with AlphaFold. *Nature* **596**, 583-589 (2021).

76. Humphrey W, Dalke A, Schulten K. VMD: visual molecular dynamics. *J Mol Graph* **14**, 33-38, 27-38 (1996).

77. Pettersen EF*, et al.* UCSF Chimera--a visualization system for exploratory research and analysis. *Journal of computational chemistry* **25**, 1605-1612 (2004).
