# Supplementary material for: Structural insights into scaffold-guided assembly of the Pseudomonas phage D3 capsid
Source: Nat Commun. 2025 Nov 23;16:11586. doi: 10.1038/s41467-025-66648-1 (PMC12749128; doi:10.1038/s41467-025-66648-1)
Supplement: Supplementary file 2 — Description of Additional Supplementary Files [file 41467_2025_66648_MOESM2_ESM.pdf]

Title: Supplementary Movie 1

Description: From Prohead 1 to Prohead 2. Comparison of surface of Prohead 1 to Prohead 2 showing that after removal of the scaffold the capsomers are subject to reshaping, notably at the 3fold interface.

Title: Supplementary Movie 2

Description: Structural transition from Prohead 1 to Prohead 2 at the pseudo-3-fold (IFG interface). Related to Fig. 7, the movie illustrates reorganization of the interface involving residue F364. The residue is proximal to the scaffold residue R102 in Prohead 1 and, following scaffold removal by proteolysis, F364 is displaced toward the center of the pseudo-3-fold, sandwiched by residues N360 and R372 in Prohead 2.

Title: Supplementary Movie 3

Description: Structural transition from Prohead 2 to the expanded head at the pseudo-3-fold (IFG interface). Related to Fig. 8, the movie illustrates the stability of the N360-F364-R372 cluster during capsid expansion.

Title: Supplementary Movie 4

Description: An animated view of 72 D3 subunits expanding shows the preservation of a salt bridge between E162 on the base of each E-loop and R219 on the backbone helix of the adjacent subunit within the same capsomer. The movie features linear morphing between models of Prohead 1, Prohead 2, and Head with pauses after each transition. Subunits are shown as ribbons; the nine D3 asymmetric unit subunits are in bold colors. E-loops are green, G-loops orange, P-loops magenta; E162 (red) and R219 (aqua) atoms are shown as space-filled spheres. Captions on the right indicate assembly stages. Prohead 1 and 2 models include placeholder residues at the E-loop tips (due to insufficient density) to facilitate morphing, though these are absent from the deposited models. The D3 MCP N-arms were not modeled in Proheads 1 or 2 and appear abruptly mid-movie in the mature capsid. Morphing was done with VMD 1.92,76 saved as DCD-format trajectories, opened in Chimera77 via MD Movie, and exported as PNG-format frames using Movie Recorder. Final editing, including captions, was done in B
