# Supplementary material for: Structural insights into scaffold-guided assembly of the Pseudomonas phage D3 capsid
Source: Nat Commun. 2025 Nov 23;16:11586. doi: 10.1038/s41467-025-66648-1 (PMC12749128; doi:10.1038/s41467-025-66648-1)
Supplement: Supplementary file 7 — Reporting Summary [file 41467_2025_66648_MOESM7_ESM.pdf]

nature portfolio

Corresponding author(s): Professor James Conway

Last updated by author(s): Nov 4, 2025

Reporting Summary

Nature Portfolio wishes to improve the reproducibility of the work that we publish. This form provides structure for consistency and transparency in reporting. For further information on Nature Portfolio policies, see our Editorial Policies and the Editorial Policy Checklist.

Statistics

For all statistical analyses, confirm that the following items are present in the figure legend, table legend, main text, or Methods section.

n/a	Confirmed	
☐	☒	The exact sample size (*n*) for each experimental group/condition, given as a discrete number and unit of measurement
☒	☐	A statement on whether measurements were taken from distinct samples or whether the same sample was measured repeatedly
☒	☐	The statistical test(s) used AND whether they are one- or two-sided<br>*Only common tests should be described solely by name; describe more complex techniques in the Methods section.*
☒	☐	A description of all covariates tested
☒	☐	A description of any assumptions or corrections, such as tests of normality and adjustment for multiple comparisons
☒	☐	A full description of the statistical parameters including central tendency (e.g. means) or other basic estimates (e.g. regression coefficient) AND variation (e.g. standard deviation) or associated estimates of uncertainty (e.g. confidence intervals)
☒	☐	For null hypothesis testing, the test statistic (e.g. *F*, *t*, *r*) with confidence intervals, effect sizes, degrees of freedom and *P* value noted<br>*Give P values as exact values whenever suitable.*
☒	☐	For Bayesian analysis, information on the choice of priors and Markov chain Monte Carlo settings
☒	☐	For hierarchical and complex designs, identification of the appropriate level for tests and full reporting of outcomes
☒	☐	Estimates of effect sizes (e.g. Cohen's *d*, Pearson's *r*), indicating how they were calculated

Our web collection on statistics for biologists contains articles on many of the points above.

Software and code

Policy information about availability of computer code

Data collection	Thermo Fisher Scientific "EPU" v3.5 and v3.7
Data analysis	MRC "RELION" v3 and v4; EMAN2 2.22; SPHIRE-crYOLO 1.78 and 1.8.2; UCSF ChimeraX 1.6-1.8; ISOLDE 1.6-1.8; MolProbity 4.5; Resmap 1.1.4

For manuscripts utilizing custom algorithms or software that are central to the research but not yet described in published literature, software must be made available to editors and reviewers. We strongly encourage code deposition in a community repository (e.g. GitHub). See the Nature Portfolio guidelines for submitting code & software for further information.

Data

Policy information about availability of data

All manuscripts must include a data availability statement. This statement should provide the following information, where applicable:

- Accession codes, unique identifiers, or web links for publicly available datasets
- A description of any restrictions on data availability
- For clinical datasets or third party data, please ensure that the statement adheres to our policy

The source data underlying Figure 2 and Supplementary Figure S1 are provided as a Source Data file.
The final cryo-EM reconstructions are deposited with the Electron Microscopy Data Bank (EMDB) under accession codes EMD-70800 [https://www.ebi.ac.uk/emdb/EMD-70800] (D3 Prohead 1 capsid), EMD-70832 [https://www.ebi.ac.uk/emdb/EMD-70832] (D3 Prohead 2 capsid), EMD-70878 [https://www.ebi.ac.uk/emdb/EMD-70878] (D3 virion capsid), EMD-70884 [https://www.ebi.ac.uk/emdb/EMD-70884] (D3 empty head capsid), EMD-70831 [https://www.ebi.ac.uk/emdb/EMD-70831] (D3 Prohead 1 vertex), EMD-70834 [https://www.ebi.ac.uk/emdb/EMD-70834] (D3 Prohead 2 vertex), EMD-70879 [https://www.ebi.ac.uk/emdb/

EMD-70879] (D3 virion vertex), and EMD-70887 [https://www.ebi.ac.uk/emdb/EMD-70887] (D3 empty head vertex), and the fitted coordinates with the Protein Data Bank (PDB) under accession codes PDB-9OSB [https://doi.org/10.2210/pdb9OSB/pdb] (D3 Prohead 1 vertex), PDB-9OTH [https://doi.org/10.2210/pdb9OTH/pdb] (D3 Prohead 2 vertex), PDB-9OUS [https://doi.org/10.2210/pdb9OUS/pdb] (D3 virion vertex), and PDB-9OUZ [https://doi.org/10.2210/pdb9OUZ/pdb] (D3 empty head vertex).

Research involving human participants, their data, or biological material

Policy information about studies with human participants or human data. See also policy information about sex, gender (identity/presentation), and sexual orientation and race, ethnicity and racism.

Reporting on sex and gender	Not applicable.
Reporting on race, ethnicity, or other socially relevant groupings	Not applicable.
Population characteristics	Not applicable.
Recruitment	Not applicable.
Ethics oversight	Not applicable.

Note that full information on the approval of the study protocol must also be provided in the manuscript.

Field-specific reporting

Please select the one below that is the best fit for your research. If you are not sure, read the appropriate sections before making your selection.

[x] Life sciences [ ] Behavioural & social sciences [ ] Ecological, evolutionary & environmental sciences

For a reference copy of the document with all sections, see nature.com/documents/nr-reporting-summary-flat.pdf

Life sciences study design

All studies must disclose on these points even when the disclosure is negative.

Sample size	For sample preparation, sample sizes were not calculated because no statistical analysis was performed. Datasets of 2972 to 43546 micrographs were collected according to particle concentrations and were used in the cryoEM analyses. Sufficiency for structural reconstitution is demonstrated in Table 1 and Supplementary Figures S1-S3.
Data exclusions	Poor quality particles were excluded, as normal during cryo-EM analyses. No other data were excluded.
Replication	Biochemical experiments were performed independently at least three times to confirm reproducibility. Cryo-EM datasets were each analysed once as each density map represents the average of thousands of particle images.
Randomization	Randomization of data in biochemical experiments is not relevent. The "gold-standard" processing pathway used by the cryoEM reconstruction software, Relion, treats the data as two independent half-sets for reducing bias when calculating resolutiuon.
Blinding	In the absence of subjective allocation, blinding was irrelevant to this study.

Reporting for specific materials, systems and methods

We require information from authors about some types of materials, experimental systems and methods used in many studies. Here, indicate whether each material, system or method listed is relevant to your study. If you are not sure if a list item applies to your research, read the appropriate section before selecting a response.

Materials & experimental systems

n/a	Involved in the study	
[x]	[ ]	Antibodies
[x]	[ ]	Eukaryotic cell lines
[x]	[ ]	Palaeontology and archaeology
[x]	[ ]	Animals and other organisms
[x]	[ ]	Clinical data
[x]	[ ]	Dual use research of concern
[x]	[ ]	Plants

Methods

n/a	Involved in the study	
[x]	[ ]	ChIP-seq
[x]	[ ]	Flow cytometry
[x]	[ ]	MRI-based neuroimaging

Plants

Seed stocks	Not applicable.
Novel plant genotypes	Not applicable.
Authentication	Not applicable.
